# Negative impact of increasing age on clinical outcomes of arthroscopic lateral ankle ligament repair for chronic lateral ankle instability

**DOI:** 10.1002/jeo2.70328

**Published:** 2025-07-07

**Authors:** Bagus Iman Brilianto, Tomoyuki Nakasa, Yasunari Ikuta, Shingo Kawabata, Dan Moriwaki, Satoru Sakurai, Saori Ishibashi, Nobuo Adachi

**Affiliations:** ^1^ Department of Orthopadic Surgery Graduate School of Biomedical and Health Sciences Hiroshima University Hiroshima Japan; ^2^ Department of Orthopaedics and Traumatology Prof. Dr. R. Soeharso Orthopaedic Hospital Surakarta Indonesia; ^3^ Department of Artificial Joints and Biomaterials Graduate School of Biomedical and Health Sciences Hiroshima University Hiroshima Japan

**Keywords:** age, ankle lateral ligament, arthroscopic, repair

## Abstract

**Purpose:**

Arthroscopic lateral ankle ligament repair is gaining popularity for chronic lateral ankle instability (CLAI). Several studies have reported excellent outcomes with this procedure. However, the effect of age on clinical outcomes remains unclear, particularly factors that may lead to poor postoperative outcomes. This study aimed to analyze the effect of age on the clinical outcomes of arthroscopic repair for CLAI.

**Methods:**

Seventy‐five ankles of 69 patients (35.6 ± 15.8 years) that underwent arthroscopic anterior talofibular ligament (ATFL) repair with calcaneofibular ligament (CFL) repair for CLAI between February 2019 and December 2023 were retrospectively reviewed. In these patients, arthroscopic ATFL repair was performed with additional CFL repair if the varus instability remained. The clinical outcomes, including the Japanese Society for Surgery of the Foot (JSSF) hindfoot‐ankle scale, Karlsson‐Peterson (K‐P) score and Self‐Administered Foot Evaluation Questionnaire (SAFE‐Q) score, were evaluated preoperatively and at the final follow‐up. The patients were classified into younger (<40 years) and older (≥40 years) groups, and the outcomes were compared.

**Results:**

The mean follow‐up period was 15.3 ± 7.2 months. The JSSF scale, K‐P and all SAFE‐Q subscale scores significantly improved postoperatively. Nineteen ankles exhibited recurrent instability (25.3%); however, no ankles required revision surgery. Age was negatively correlated with postoperative clinical scores. K‐P score was significantly lower in the older group than in the younger group preoperatively. Postoperatively, the JSSF scale, K‐P score, pain and pain‐related, and general health and well‐being on the SAFE‐Q subscale were significantly lower in the older group than in the younger group. Multiple regression analysis revealed a significant association of age, preoperative JSSF scale, and SAFE‐Q preoperative social functioning and general health and well‐being subscales with postoperative JSSF scale.

**Conclusions:**

Increasing age negatively affects the clinical outcomes of arthroscopic repair for CLAI due to residual pain and poor mental health.

**Level of Evidence:**

Level IV, retrospective case series.

**Abbreviations**
AAL: accessory anterolateral portal
AAS: ankle activity score
AL: anterolateral
ATFL: anterior talofibular ligament
CFL: calcaneofibular ligament
CLAI: chronic lateral ankle instability
JSSF: Japanese Society for Surgery of the Foot
K‐P: Karlsson‐Peterson
OA: osteoarthritis
SAFE‐Q: Self‐Administered Foot Evaluation Questionnaire

## INTRODUCTION

Ankle sprains are among the most common injuries, particularly during physical activity. More than 75% of acute ankle sprains are lateral injuries to the anterior talofibular ligament (ATFL), followed by injuries to the calcaneofibular ligament (CFL) [1, 2]. Approximately 80% of acute ankle sprains are successfully treated with conservative management; however, 10%–30% of patients with multiple lateral ankle sprains show progression to chronic lateral ankle instability (CLAI) [34]. This condition can cause dysfunction and pain due to ankle instability, leading to impairments in daily living [19]. Furthermore, CLAI can progress to early osteoarthritis (OA) because of uneven high‐stress distribution in the ankle and subtalar joints [33]. Therefore, surgical correction for instability is performed in younger, athletic and older patients to prevent the development of OA and improve their daily living activities.

The arthroscopic ATFL repair procedure has recently become popular, with reports of favourable clinical outcomes [4, 32]. This minimally invasive procedure preserves the soft tissue of the ankle joint, offering a faster recovery to sports or daily activities compared with open surgery [9, 15]. Although arthroscopic lateral ankle ligament repair is performed in patients of various ages to return to the pre‐injury level of sports or improve daily activities, the effect of age on the clinical outcomes of this procedure remains unclear. In older patients, postoperative outcomes may be worse than those in younger patients because of slower muscle recovery and deterioration of the ATFL remnant and anterolateral (AL) joint capsule condition due to the long period between injury and surgery. It was hypothesized that older patients would have worse postoperative outcomes than younger patients and that several characteristics would result in worse outcomes in these older patients. In most arthroscopic lateral ankle ligament repairs, the functional status of the CFL remains uncertain, posing the risk of poor outcomes due to residual CFL dysfunction [31]. Isolated ATFL repair in ankles with ATFL and CFL deficiencies may result in residual instability after surgery, affecting the evaluation of postoperative outcomes [14]. To accurately evaluate the effect of age on postoperative outcomes, ATFL and CFL dysfunctions should be resolved immediately after surgery. This study aimed to evaluate the effects of age on clinical outcomes of arthroscopic ATFL repair with CFL repair for CLAI. The primary outcomes were to elucidate whether increasing age negatively affected clinical outcomes, and the secondary outcomes were to identify factors associated with worse clinical outcomes in older patients.

## MATERIALS AND METHODS

### Participants

Between February 2019 and December 2023, 162 ankles of 148 patients diagnosed with CLAI and treated surgically were retrospectively reviewed. The inclusion criteria were arthroscopic ligament repair with a minimum 1‐year follow‐up period, complete preoperative Self‐Administered Foot Evaluation Questionnaire (SAFE‐Q) data, and preoperative computed tomography and magnetic resonance imaging. Patients with systemic diseases, such as rheumatoid arthritis, revision surgery, chondral/osteochondral lesions, subfibular ossicles greater than 5 mm, abnormal hindfoot alignment, generalized ligament laxity and OA with a Takakura‐Tanaka classification of grade 2 or higher were excluded [30]. Finally, 75 ankles of 69 patients (32 men and 37 women; mean age, 35.6 ± 15.8 [range: 14–66] years) were eligible for this study. Six patients had bilateral ankle involvement. This study was approved by the local ethics committee (approval number: E‐879), and written informed consent was obtained from all eligible participants.

### Surgical technique

**Figure 1:**
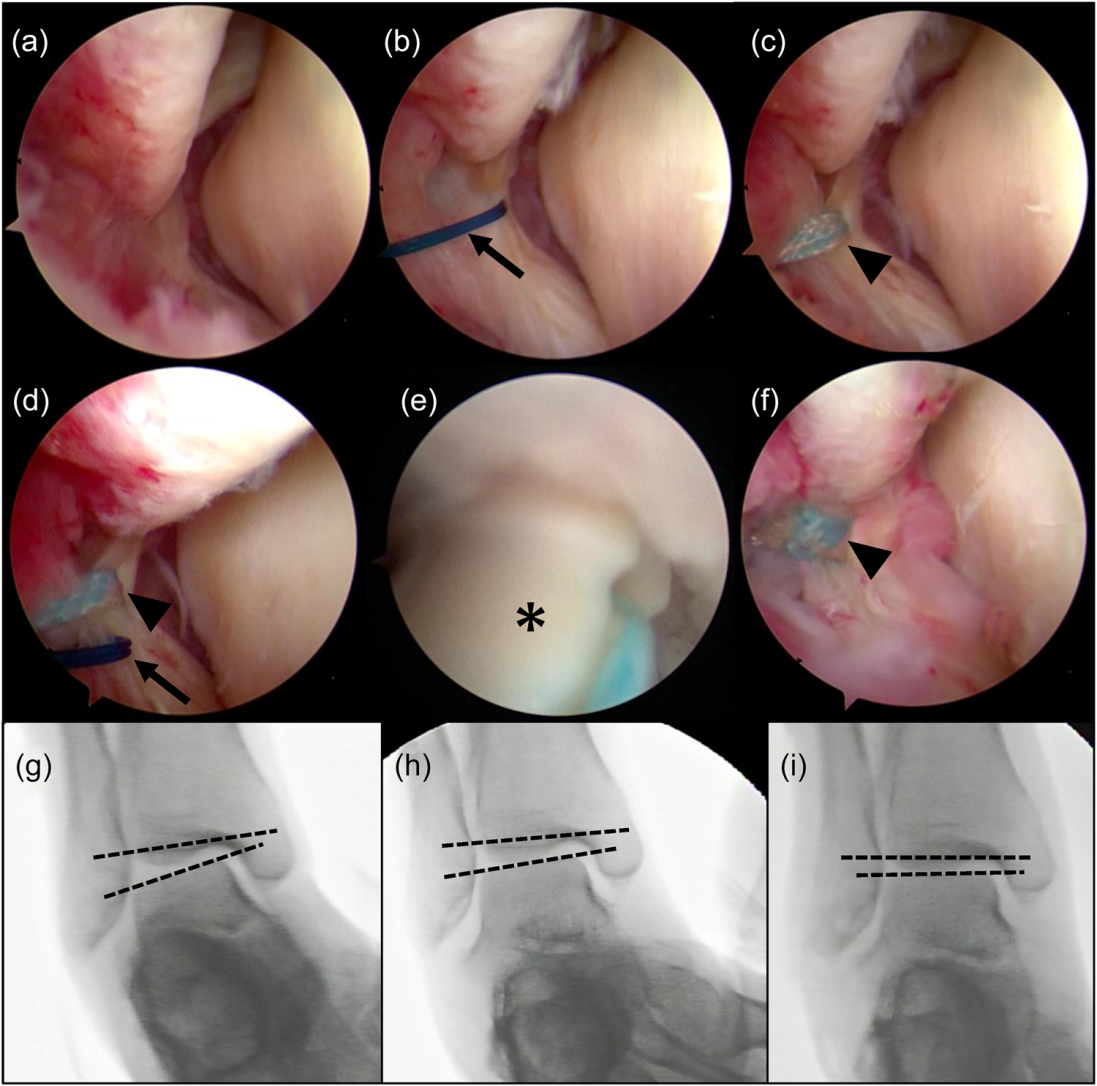
Arthroscopic ATFL repair procedure. (a) Observation of the ATFL remnant via the anterolateral portal. (b) A 3‐0 nylon thread was inserted to penetrate the proximal third of the ATFL remnant. (c) Suture tape was introduced using a 3‐0 nylon loop and sewn using a racking hitch knot. (d) Additional knots were placed. (e) A knotless anchor was inserted at the footprint of the ATFL. (f) After anchor fixation. (g) Preoperative varus stress. (h) Immediately after the ATFL repair. The CFL repair was performed if residual instability was observed. (i) After the ATFL and CFL repair. Arrow indicates 3‐0 nylon loop. Arrowhead indicates suture tape. *Knotless anchor. ATFL, anterior talofibular ligament; CFL, calcaneofibular ligament.

The arthroscopic ATFL repair combined with the additional CFL repair was performed as previously described [13, 20]. Standard AL and anteromedial portals were created, and a 30‐degree oblique 2.7 mm arthroscope was inserted. Following the evaluation of the ankle joint, an accessory AL portal (AAL) was placed along the anterior border of the fibula as the working portal, and the AL portal was used as the viewing portal. A 23‐G hollow needle with a 3‐0 nylon thread was inserted to penetrate the proximal third of the ATFL remnant. The nylon loop was retrieved using a mosquito clamp, and the needle was withdrawn. Subsequently, a No. 2 thread (Ultra‐braid; Smith & Nephew) or suture tape (Ultra‐tape; Smith & Nephew) was passed through a nylon loop and sewn using a racking hitch knot (Figure 1). Two additional knots were placed at the ATFL remnant, on both sides of the first knot. A hole was drilled into the ATFL footprint in the fibula. A knotless anchor (Bioraptor Knotless; Smith & Nephew) with a No. 2 thread or suture tape was inserted and tightened to the footprint site, with the ankle joint in a neutral position. Thereafter, a varus stress test under fluoroscopy was manually performed to confirm the CFL deficiency. If the talar tilting angle (TTA) remained after ATFL repair, a CFL repair was performed. The AAL portal was extended approximately 15 mm toward the fibular tip, exposing the CFL. From this incision, the connection between the CFL and ATFL inferior fascicle was confirmed. A soft anchor (Q‐Fix Mini; Smith & Nephew) was inserted into the CFL footprint under direct visualization. One end of the anchor suture was passed into the fibular side of the CFL and tied using the sliding knot technique. A varus stress test was performed again after CFL repair to re‐evaluate the TTA. Postoperatively, the ankle joint was immobilized using a casting system with a cover (FitCure‐Ankle; Alcare Co. Ltd.) to restrict the ankle motion for 4 weeks postoperatively. Weightbearing was permitted the day after surgery. Walking, squats and intrinsic foot muscle training were initiated for up to 2 weeks. After 2 weeks, dorsiflexion and plantarflexion were permitted, and proprioceptive training was initiated. Jogging was permitted 6 weeks postoperatively.

### Arthroscopic evaluation of the ATFL remnant

The ATFL remnant was observed through the AL portal, and classified into three groups as described previously [18]: (1) poor, arthroscopic observation of a clearly hypoplastic ligament with poorly defined margins; (2) moderate, arthroscopic observation of fibrotic tissue or synovitis demonstrating a stretched hyperplastic or hypoplastic ligament; and (3) excellent, arthroscopic observation of normal synovial tissue demonstrating a ligament with sharply defined margins.

### Clinical evaluation

The clinical outcomes were evaluated preoperatively and at the final follow‐up using the Japanese Society for Surgery of the Foot (JSSF) hindfoot‐ankle scale [22, 23], Karlsson‐Peterson (K‐P) scoring system [10] and patient‐reported outcomes using the SAFE‐Q, comprising five subscales: (1) pain and pain‐related; (2) physical functioning; (3) social functioning; (4) shoe‐related and (5) general health and well‐being [24, 25]. The preoperative activity was assessed using the ankle activity score (AAS), which comprised 53 sports, working activities, and 4 general activities [8]. Varus stress radiography was performed with a force of 50 N using a tension device (Imada) preoperatively and 1 year postoperatively, and the TTA was measured. The experiences of ankle sprain after surgery or TTA of ≥6° at 1 year postoperatively were defined as recurrent instability as described previously [21]. The patients were divided into older (≥40 years) and younger (≤39 years) groups because of the decline in physical functions after the age of 40 years, and their data were compared [6].

### Statistical analysis

Paired *t*‐tests were used to compare the JSSF scale, K‐P score, SAFE‐Q and TTA before and after surgery. The Mann–Whitney *U*‐test was used to compare the parameters between the older and younger groups. Spearman's correlation coefficient (*r*_s_) was used to explore the relationship between the two groups. For absolute values of *r*_s_, 0–0.19 was regarded as very weak, 0.2–0.39 as weak, 0.40–0.59 as moderate, 0.6–0.79 as strong and 0.8–1 as very strong correlation. Post hoc power analysis was performed using G*Power. Sample sizes of 38 in the younger group and 37 in the older group were used for the statistical power analysis. The effect sizes used for this assessment were large (*d* = 0.8), and the alpha level used was *p* < 0.05. The post hoc analysis revealed that the statistical power for this study was 0.92. Thus, there was adequate power at the large effect size level. Stepwise multiple regression analysis was performed to detect the factors that significantly influenced the postoperative JSSF scale. The independent variables included age, sex, body mass index (BMI), preoperative JSSF scale, preoperative TTA and all five subscales of preoperative SAFE‐Q. Multicollinearity was determined based on the variance inflation factor (VIF), with multicollinearity identified at VIF ≥ 10. The adjusted *R*‐squared value was used to measure the goodness of fit of the linear regression model. All analyses were performed using JMP Pro, version 18.0.1 (JMP Statistical Discovery LLC), and statistical significance was set at *p* < 0.05. The minimal clinically important differences (MCID) for the JSSF scale and the subscales of the SAFE‐Q were determined using a distribution‐based method [17]. The MCID for each scale was calculated as one‐half the standard deviation (0.5 SD) of the mean change score between preoperative and postoperative assessments for all patients. The proportion of patients who achieved the MCID was evaluated.

## RESULTS

**Figure 2:**
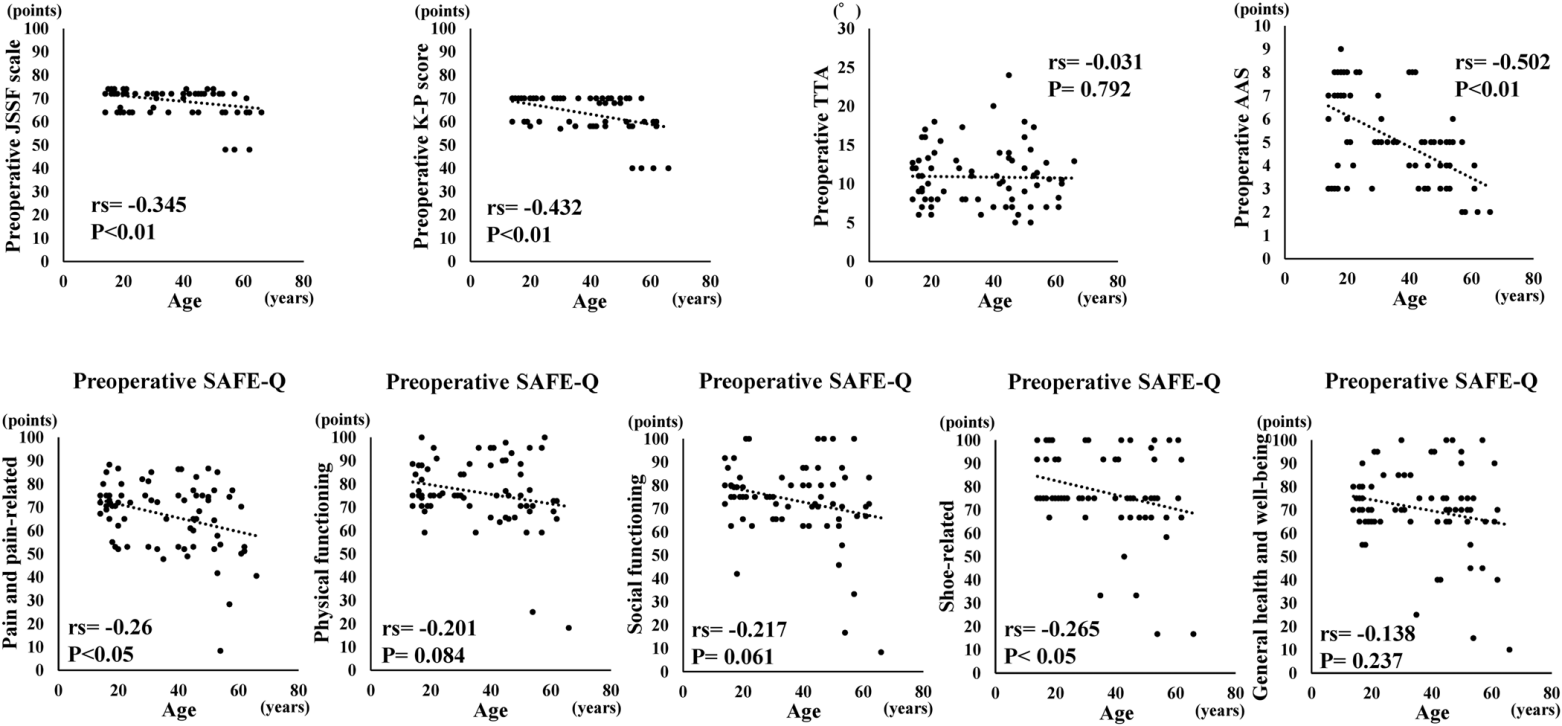
Correlations of age with preoperative parameters.

**Figure 3:**
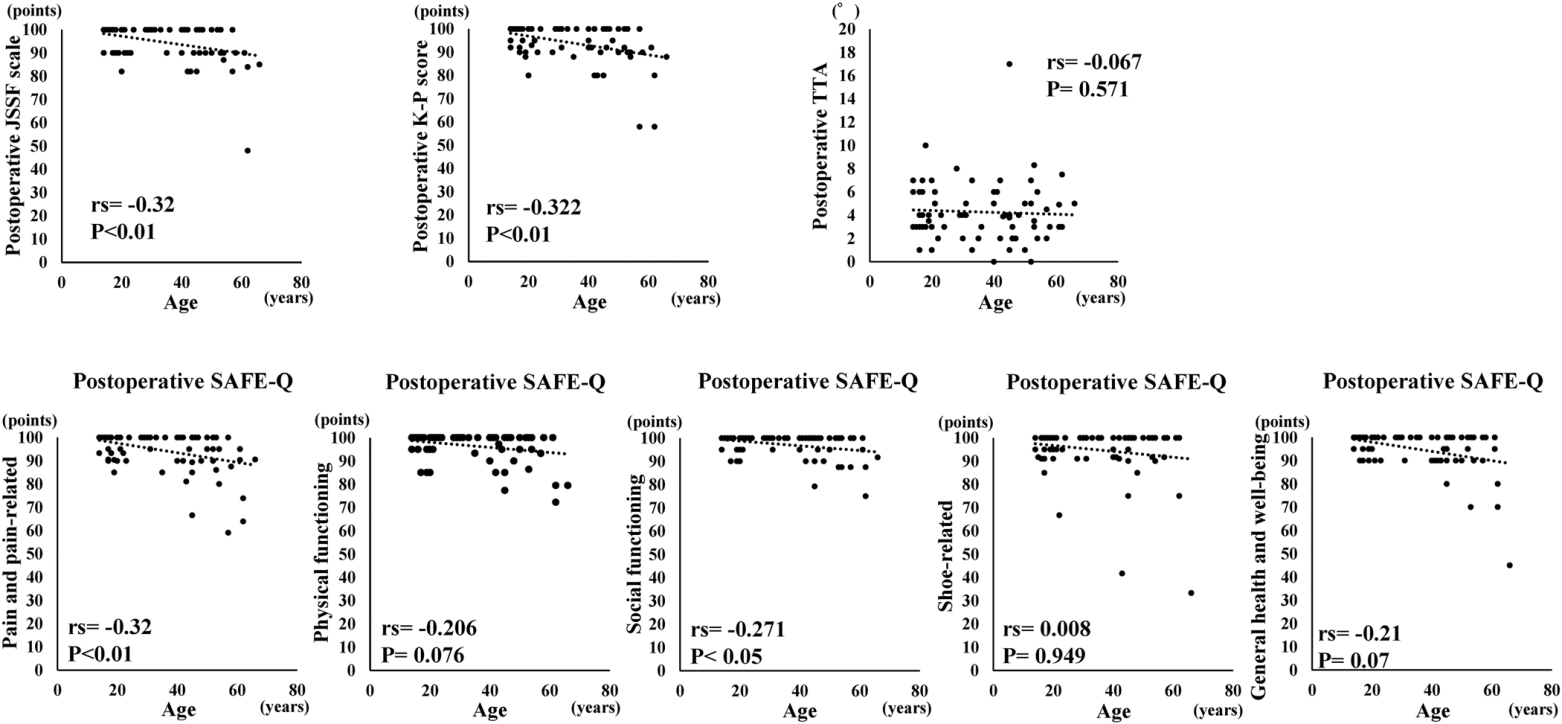
Correlations of age with postoperative parameters.

The mean follow‐up period was 15.3 ± 7.2 (range: 12–48) months. The JSSF scale, K‐P score, all subscales in the SAFE‐Q and TTA significantly improved postoperatively (Table 1). Nineteen ankles exhibited recurrent instability. All these ankles had a TAA of ≥6° (7.6 ± 2.6 [range: 6–17] degrees) at 1 year postoperatively, and 16 of the 19 ankles had experienced re‐sprains. However, no ankle required reoperation due to recurrent instability. Preoperatively, age exhibited significantly weak negative correlations with the JSSF scale, subscales of pain and pain‐related, and shoe‐related in the SAFE‐Q, and there were significant moderate negative correlations between age and the K‐P score and AAS, which means that these scores get worse with age preoperatively (Figure 2). Postoperatively, age exhibited significant negative correlations with the JSSF scale, K‐P score, and the subscales of the pain and pain‐related and social functioning in the SAFE‐Q (Figure 3). Additional CFL repair was required in 31 of 75 ankles. There were no significant differences in age, BMI and preoperative scores between the ankles with and without CFL repair. Postoperatively, no significant differences were observed in the JSSF scale and K‐P score between the ankles with and without CFL repair. However, the pain and pain‐related score in the ankles with CFL repair (97.0 ± 4.9 points) was significantly higher than in the ankles without CFL repair (92.4 ± 10.3 points) (*p* < 0.05). Regarding the remnant quality, excellent was 21 ankles, moderate 34 ankles and poor 20 ankles. Age, BMI, preoperative and postoperative scores were not significantly different in all three groups. Additional CFL repair was performed in 19.1% of the ankles with excellent remnants, 41.2% of moderate remnants and 65% of poor remnants.

**Table 1:** Preoperative and postoperative parameters.

	Preoperative	Postoperative	*p* value
JSSF scale (points)	69.3 ± 5.7 (48–74)	94.4 ± 8.4 (48–100)	<0.00001
K‐P score (points)	64.2 ± 7.8 (40–70)	93.8 ± 8.7 (58–100)	<0.00001
SAFE‐Q (points)			
Pain and pain‐related	66.7 ± 14.4 (8.3–88.3)	94.3 ± 8.7 (59.1–100)	<0.00001
Physical functioning and daily living	76.5 ± 14.1 (18.2–100)	96.4 ± 6.4 (72.3–100)	<0.00001
Social functioning	74.0 ± 16.2 (8.3–100)	97.2 ± 5.2 (75–100)	<0.00001
Shoe‐related	77.8 ± 17.7 (16.7–100)	94.7 ± 11.5 (33.3–100)	<0.00001
General health and well‐being	70.1 ± 19.2 (10–100)	94.9 ± 8.8 (45–100)	<0.00001
TTA (°)	10.9 ± 3.8 (5–24)	4.3 ± 2.6 (0–17)	<0.00001

Abbreviations: JSSF, Japanese Society for Surgery of the Foot; K‐P, Karlsson‐Peterson; SAFE‐Q, Self‐Administered Foot Evaluation Questionnaire; TTA, talar tilting angle.

No significant differences in the JSSF scale, all subscales in the SAFE‐Q and TTA were observed between the younger and older groups. The preoperative K‐P score and AAS in the older group were significantly lower than those in the younger group. Furthermore, the JSSF scale, K‐P score, subscales of pain and pain‐related and general health and well‐being were significantly lower in the older group than in the younger group preoperatively (Table 2). Recurrent instability occurred in 11 (28.9%) and 8 (21.6%) of the 38 and 37 ankles in the younger and older groups, respectively. In the younger group, isolated ATFL repair was performed for 24 (63.2%) ankles, whereas ATFL and additional CFL repair were required for 14 (36.8%) ankles. In the older group, isolated ATFL repair was performed for 20 (54.1%) ankles, whereas ATFL and CFL repair were required for 17 (45.9%) ankles. Regarding the remnant quality, the proportions of excellent, moderate and poor qualities were 34.2%, 34.2% and 31.6%, respectively, in the younger group, whereas 21.6%, 56.8% and 21.6%, respectively, in the older group.

**Table 2:** Comparison of parameters between the younger and older groups.

	Younger (*n* = 38)	Older (*n* = 37)	*p* value
Age (years)	21.6 ± 6.6 (14–36)	50.1 ± 7.0 (40–66)	
Male:female	17:21	17:20	
BMI (kg/m^2^)	23.1 ± 3.4 (17.2–31.3)	25.1 ± 3.2 (17.9–34.0)	0.124
F/U period (months)	14.4 ± 5.2 (12–33)	16.1 ± 8.8 (12–48)	0.719
Preoperative			
JSSF scale (points)	70.4 ± 3.7 (64–74)	68.2 ± 7.0 (48–74)	0.159
K‐P score (points)	66.6 ± 5.1 (58–70)	61.7 ± 9.2 (40–70)	<0.01
SAFE‐Q (points)			
Pain and pain‐related	69.9 ± 10.5 (47.7–88.3)	63.5 ± 17.1 (40.5–86.6)	0.174
Physical functioning and daily living	78.3 ± 9.5 (59.1–100)	74.5 ± 17.5 (18.2–100)	0.303
Social functioning	76.1 ± 10.4 (62.5–100)	71.7 ± 20.4 (8.3–100)	0.412
Shoe‐related	81.4 ± 13.8 (33.3–100)	74.2 ± 20.6 (16.7–100)	0.054
General health and well‐being	71.7 ± 16.9 (25–100)	68.4 ± 21.4 (10–100)	0.472
AAS (points)	6.0 ± 1.8 (3–9)	4.2 ± 1.7 (2–8)	<0.001
TTA (°)	10.8 ± 3.4 (6–17.3)	11.0 ± 4.2 (5–24)	0.976
Postoperative			
JSSF scale (points)	96.9 ± 5.1 (82–100)	91.8 ± 10.3 (48–100)	<0.05
K‐P score (points)	96.6 ± 5.1 (80–100)	90.9 ± 10.7 (58–100)	<0.05
SAFE‐Q (points)			
Pain and pain‐related	97.0 ± 4.6 (85–100)	91.6 ± 10.9 (59.1–100)	<0.05
Physical functioning and daily living	97.9 ± 4.3 (85–100)	94.8 ± 7.8 (72.3–100)	0.134
Social functioning	98.6 ± 3.1 (90–100)	95.7 ± 6.4 (72.3–100)	0.083
Shoe‐related	96.5 ± 6.4 (66.7–100)	92.9 ± 15 (33.3–100)	0.67
General health and well‐being	97.4 ± 4.0 (90–100)	92.3 ± 11.3 (45–100)	<0.05
TTA (°)	4.3 ± 2.2 (1–10)	4.2 ± 3.0 (0–17)	0.704

Abbreviations: AAS, ankle activity score; BMI, body mass index; JSSF, Japanese Society for Surgery of the Foot; K‐P, Karlsson‐Peterson; TTA, talar tilting angle.

Multiple regression analysis revealed that age, preoperative JSSF scale score, and preoperative social and general subscales on SAFE‐Q were significantly associated with the postoperative JSSF scale (Table 3). Multicollinearity was not observed among the independent variables. The adjusted *R*‐squared value was 0.51. The postoperative JSSF scale (*Y*) was correlated with age (*X*1), preoperative JSSF scale (*X*2), and preoperative social (*X*3) and general (*X*4) subscales on SAFE‐Q, and these results yielded the following equation: *Y* = 42.397 + −0.106 × 1 + 0.861 × 2 + −0.23 × 3 + 0.188 × 4. MCIDs of the JSSF scales and the subscales of the SAFE‐Q are shown in Table 4.

**Table 3:** Multiple regression analysis for postoperative JSSF scale.

	*B*	*β*	*p* value	95% CI	
Variables				Lower	Upper
Constant	42.397		<0.0001	22.88	61.91
Age	−0.106	−0.199	0.0267	−0.199	−0.013
Pre JSSF scale	0.861	0.581	<0.0001	0.573	1.149
Pre SAFE‐Q social functioning	−0.23	−0.442	0.0007	−0.36	−0.101
Pre SAFE‐Q general health and well‐being	0.188	0.428	0.001	0.0789	0.297

Abbreviations: *B*, partial regression coefficient; *β*, standardized partial regression coefficient; CI, confidence interval; JSSF, Japanese Society for Surgery of the Foot; SAFE‐Q, Self‐Administered Foot Evaluation Questionnaire.

**Table 4:** Minimal clinically important difference (MCID) values in the younger and older groups.

	MCID	Score changes over MCID (%)	
		Younger group	Older group
JSSF scale (points)	3.2	100% (38/38)	97.3% (36/37)
SAFE‐Q (points)			
Pain and pain‐related	6.3	97.4 (37/38)	94.6 (35/37)
Physical functioning and daily living	7.1	89.5 (34/38)	70.3 (26/37)
Social functioning	7.8	94.7 (36/38)	78.4 (29/37)
Shoe‐related	8.9	60.5 (23/38)	64.9 (24/37)
General health and well‐being	9.3	86.8 (33/38)	78.4 (29/37)

*Note*: Score changes were defined as the differences in each scale between preoperative and postoperative assessments.

Abbreviations: JSSF, Japanese Society for Surgery of the Foot; SAFE‐Q, Self‐Administered Foot Evaluation Questionnaire.

## DISCUSSION

This study revealed that increasing age negatively affected clinical outcomes of the arthroscopic ATFL repair with additional CFL repair, which is the primary outcome of this study. The secondary outcomes of this study were to identify factors of worse clinical outcomes in older patients, and older patients exhibited lower JSSF and K‐P scores, and the subscales of the pain and pain‐related and social functioning in the SAFE‐Q, which means that older patients were more anxious and feeling depressed postoperatively and had more residual pain than younger patients.

Many studies on arthroscopic lateral ankle ligament repair, including older patients with CLAI, have been reported, as it is a common surgery for the general population and not just athletes [26, 27, 39]. Although several risk factors, such as remnant quality, high BMI, varus tibia plafond, and general laxity, for poor clinical outcomes, including recurrent instability, have been reported, the impact of increasing age on outcomes and causes remains unknown [20, 38, 39, 40]. Chen et al. [3] reported that postoperative immobilization with a lower cast, female sex, non‐sports‐related ankle sprain and increasing age were significant predictors of poorer Karlsson Ankle Functional Score in arthroscopic ATFL repair. Li et al. [12] demonstrated a negative correlation between the Karlsson Ankle Functional Score and Tegner activity score of arthroscopic ATFL repair with age. Likewise, a correlation between increasing age and poor postoperative scores was found in this study. Furthermore, the current study revealed higher complaints of increased residual pain and feeling anxious and depressed postoperatively in older patients, which can cause worse postoperative outcomes after arthroscopic lateral ankle ligament repair.

Some ankles with CLAI have ATFL and CFL injuries. CFL dysfunction may also increase with age in patients with CLAI, as persistent ATFL dysfunction exerts a high deforming force on the CFL [28, 35]. Most studies evaluating the postoperative outcomes of arthroscopic ATFL repair have not accounted for CFL dysfunction. A cadaveric study revealed that isolated ATFL repair could make the CFL stump contact its footprint; however, the CFL did not exhibit a normal strain pattern [31]. Since the surgery was completed after confirming the absence of CFL dysfunction, there should be no CFL dysfunction immediately after surgery in the current study. The exclusion of the effect of CFL dysfunction after ATFL repair has the advantage of assessing the effect of age on postoperative outcomes. Furthermore, a multiple regression analysis in the current study revealed that the preoperative subscales of the SAFE‐Q, social functioning, and general health and well‐being were associated with the postoperative JSSF scale. The subscale of social functioning evaluates the extent to which the activities of daily living, such as work, school and leisure, can be performed [25]. A low preoperative social functioning score indicates potential impairment of motor function in patients because of the significant loss of muscle strength, making it difficult for them to perform daily activities. Preoperatively, older patients had significantly lower K‐P scores and AAS than younger patients. The K‐P score emphasizes the activities of daily living more than the JSSF scale, as the K‐P score evaluates pain, swelling, instability, stiffness, stair climbing, running, work activities and support. Many patients with CLAI experience pain and are less active, specifically the older patients in the current study. Therefore, restoring motor function may be difficult, even if surgery improves the instability. The subscale of general health and well‐being evaluates mental conditions, such as feeling anxious, depressed and frustrated due to ankle symptoms. The scores for this subscale were significantly lower postoperatively in the older group than in the younger group. Psychosocial problems are reportedly associated with low satisfaction after surgery [5, 7, 16, 36]. Previous reports demonstrated that depression in older patients decreased clinical outcomes after surgery [29, 37]. Therefore, mental health care may be necessary to achieve high postoperative satisfaction in older patients after surgery.

Recent studies have widely reported an association between poor remnant quality and poor postoperative outcomes, including recurrent instability [13, 39]. Notably, loosening of the AL capsule causes poor clinical outcomes due to recurrent instability [21]. The current study showed no significant differences in postoperative outcomes by remnant quality. Additional CFL repair was performed more frequently in the poor remnant ankles, and it may augment the poor ATFL remnant, resulting in no difference in postoperative outcomes. In addition, the older group had fewer excellent remnants than the younger group. In older patients, the time between injury and surgery may be longer, degeneration of the ATFL remnant and AL capsule may be more advanced, and arthroscopic repair may not provide adequate tension to eliminate ankle instability. Additionally, the postoperative recurrent instability rate was higher in the younger group in this study. Previous reports showed that the quality of the ATFL remnant is often poor in CLAI patients with high activity, and a poor ATFL remnant is a risk factor for recurrent instability [13, 39]. Since the older group was less active than the younger group, the rate of poor ATFL remnant and recurrent instability may be lower than in the younger group. Nevertheless, the worse score for the older group may not be due to the quality of the remnant, but rather to the cause of the pain and the mental condition of the patients postoperatively. A previous study reported that pain in the CLAI causes synovitis or subchondral bone sclerosis in the medial gutter, except in chondral/osteochondral lesions [19]. In older patients, the duration of CLAI is longer, and pain due to osteosclerosis of the medial gutter may persist postoperatively. In addition, older patients may have more functional impairments than younger patients because of muscle weakness, specifically in the peroneal muscles, which may cause worse postoperative outcomes. The results of this study suggest that it is important to educate the public and promote earlier lateral ligament stabilization for symptomatic CLAI, which leads to better clinical outcomes with lesser pain before irreversible changes, such as subchondral bone sclerosis or cartilage injuries.

This study has some limitations. First, the sample size was small. Many exclusion criteria to eliminate influences other than age resulted in a small number of patients being included. Therefore, patients were divided into younger and older groups based on an age cutoff of 40 years. However, MCID values of the JSSF scale and the subscales of the SAFE‐Q are sufficient to discuss the results of this study. Nevertheless, the results may differ between patients in their 40s and 60s, even within the older group. Consequently, larger populations should be evaluated for each age group. Second, all patients' preoperative and postoperative muscle strengths were not measured. Age‐related losses of muscle mass and strength may influence the speed and quality of recovery postoperatively. Finally, the follow‐up period was short. Due to the slow recovery of older patients postoperatively, it remains unclear whether their long‐term clinical outcomes differ from those of younger patients. Studies with a longer follow‐up duration and larger sample size might provide more information about the effect of age on the outcomes of arthroscopic ATFL repair.

## CONCLUSION

Older patients have worse clinical outcomes of arthroscopic lateral ankle ligament repair than younger patients. Older age decreases subscales related to pain and mental conditions, resulting in worse postoperative outcomes in arthroscopic ligament repair for CLAI. It is important to promote earlier lateral ligament stabilization for symptomatic CLAI for better clinical outcomes with less pain.

## AUTHOR CONTRIBUTIONS

**Bagus Iman Brilianto**: Methodology; formal analysis and investigation; writing—original draft preparation. **Tomoyuki Nakasa**: Conceptualization; methodology; writing—review and editing. **Yasunari Ikuta**: Writing—original draft preparation. **Shingo Kawabata**: Formal analysis and investigation. **Dan Moriwaki**: Formal analysis and investigation. **Satoru Sakurai**: Formal analysis and investigation. **Saori Ishibashi**: Formal analysis and investigation. **Nobuo Adachi**: Supervision.

## CONFLICT OF INTEREST STATEMENT

The authors declare no conflicts of interest.

## ETHICS STATEMENT

The study was approved by the local ethics committee of our university. Written informed consent was obtained from all participants.

## Data Availability

The data sets used and/or analyzed during the current study are available from the corresponding author on reasonable request.
